# Mu-8: visualizing differences between proteins and their families

**DOI:** 10.1186/1753-6561-8-S2-S5

**Published:** 2014-08-28

**Authors:** Johnathan D Mercer, Balaji Pandian, Alexander Lex, Nicolas Bonneel, Hanspeter Pfister

**Affiliations:** 1 Harvard University, 33 Oxford Street, MA 02138 Cambridge, USA; 2 Broad Institute, 7 Cambridge Center, MA 02142 Cambridge, USA

## Abstract

**Background:**

A complete understanding of the relationship between the amino acid sequence and resulting protein function remains an open problem in the biophysical sciences. Current approaches often rely on diagnosing functionally relevant mutations by determining whether an amino acid frequently occurs at a specific position within the protein family. However, these methods do not account for the biophysical properties and the 3D structure of the protein. We have developed an interactive visualization technique, Mu-8, that provides researchers with a holistic view of the differences of a selected protein with respect to a family of homologous proteins. Mu-8 helps to identify areas of the protein that exhibit: (1) significantly different bio-chemical characteristics, (2) relative conservation in the family, and (3) proximity to other regions that have suspect behavior in the folded protein.

**Methods:**

Our approach quantifies and communicates the difference between a reference protein and its family based on amino acid indices or principal components of amino acid index classes, while accounting for conservation, proximity amongst residues, and overall 3D structure.

**Results:**

We demonstrate Mu-8 in a case study with data provided by the 2013 BioVis contest. When comparing the sequence of a dysfunctional protein to its functional family, Mu-8 reveals several candidate regions that may cause function to break down.

## Introduction

Proteins are commonly known as the "workhorse" macromolecules that perform vital cellular and extracellular functions in an organism. Their roles include, but are not limited to catalysis of biochemical reactions, transportation, storage, and communication. A protein is made of a sequence of amino acids (also referred to as residues) that are coded for by genes. A protein derives its function from its three-dimensional structure (the tertiary structure), which is in turn driven by the biochemical properties of its amino acid sequence (the primary structure). Understanding and being able to predict the 3D structure from the amino acid sequence, however, is part of the unsolved protein-folding problem [1].

While a general solution to this problem is not within reach of current methods, interactive visualization and computational analysis can help biologists understand the relationship between the amino acid sequence and a protein's 3D structure. This in turn will facilitate the analysis of protein function.

Motivated by the problem and the data published for the 2013 IEEE BioVis Data Contest [2], we developed *Mu-8*, a novel, interactive visualization tool for comparing a *reference protein *to a large protein family. Mu-8 can be accessed at http://mu-8.com. Different or altered proteins often fulfill the same function, albeit with different efficiency. Such proteins are referred to as a protein family and are mostly evolutionary related. This demonstrates that function is often preserved even if the amino acid sequence is changed. On the other hand, small changes to the sequence can sometimes cause function to break down. Mu-8 was designed to identify which mutation(s) in a highly mutated amino acid sequence cause a functional break-down. Using Mu-8, we are able to: (1) quickly identify residues or regions of residues that are significantly different from the family with respect to one or more characteristics; (2) identify whether such a region is in an otherwise highly conserved area of the sequence; and (3) assess the spatial relationships to other regions of the sequence.

**Figure 1:**
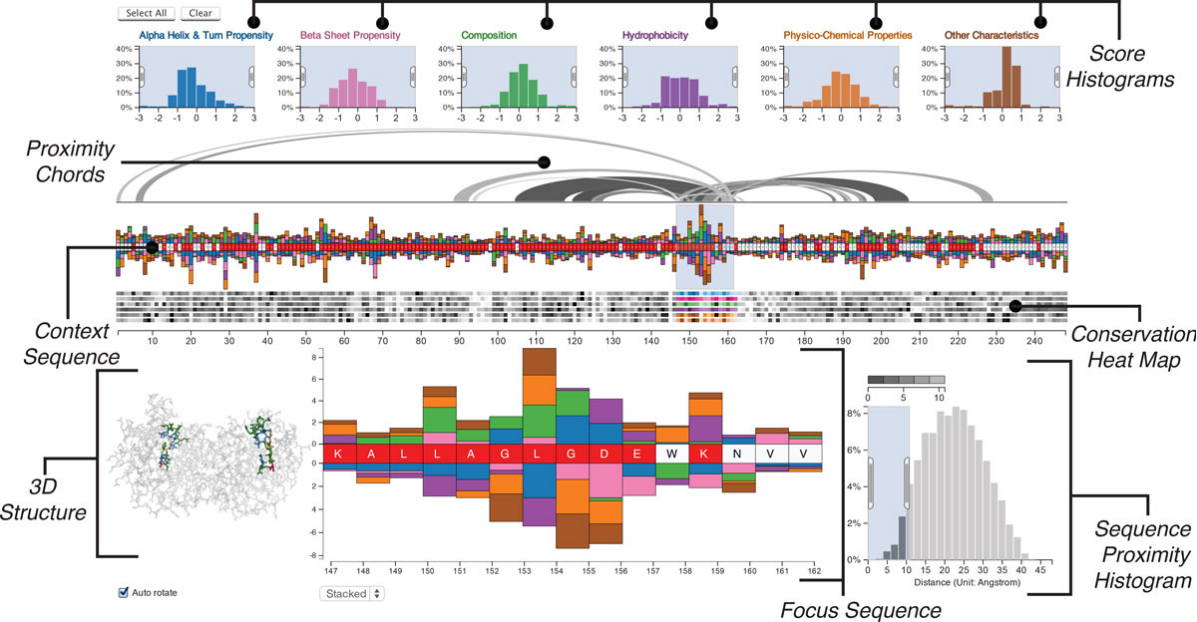
**The annotated Mu-8 interface showing how characteristics of a defective protein compare to its functional family**.

We demonstrate the value of Mu-8 on the dataset published by the BioVis Data Contest, where we identify several regions of interest. Most notable are the residues at positions 150-156, which mutated from "VLEEVKD" to "LAGLGDE", shown in the focus region in Figure 1. These residues are significantly different from the family across many biophysical properties, are located in relatively conserved regions, and are close to other regions with similar anomalies in the folded protein. This region is also close to the protein's active site as *lysine *12, *histidine *95, and *glutamic acid *165 are directly involved in the metabolic process [3]. It stands to reason that the mutated region 150-156 may have contorted the location and orientation of the active site, thus rendering the protein dysfunctional.

## Concept

Our design strategy was predicated on basic principles that we elicited in interviews with domain experts and an extensive literature review. First, we required a design that focuses on the differences of a defective protein to its functional family, without having to show every family member. Second, we required both a holistic view of the sequence and the differences to the family. Finally, we aimed at closely coupling the analysis of the sequence with the inspection of the 3D structure.

To measure how different a residue in the mutated protein is compared to the protein family we use amino acid indices, which are an invaluable resource for judging the potential consequence of a mutation. An amino acid index is a quantitative score assigned to each of the amino acids. They predict various biophysical properties and their development has become a mainstay in protein research pioneered by Chou and Fasman [4].

However, there are hundreds of amino acid indices, and determining which of them are relevant to the loss of function is difficult. At the same time, showing all indices in a visualization is a challenge with respect to scalability and introduces significant complexity. While attempts have been made at correlating these indices together to provide a lower-dimensional representation [5], and correlating them with structural properties [6,7], this comes at the price of discarding information that can remain relevant for our task. To address this problem we offer two options: analysts can use a single representative score for each of the amino acid's six major characteristics (the default option), or they can choose which amino acid indices to consider.

Our approach is based on the assumption that significantly different characteristics of substituted amino acids are more likely to cause functional changes. Consequently, we visualize a score, which we call the *c-score*, that quantifies how "different" a characteristic of an amino acid of the reference protein is from its family. Furthermore, mutations affecting function often occur in otherwise conserved regions, i.e., regions with low variation of residues in homologous proteins, due to evolutionary selection for functional proteins. Our scores also account for this variation in the family. The distribution of these c-scores are shown in the *Score Histograms*, while the individual scores for each amino acid are shown as bars in the *Context Sequence *view and, in more detail, in the *Focus Sequence *view (see Figure 1). To complement these scores we also highlight conserved regions with a *Conservation Heat Map*, also shown in Figure 1, which shows the variation of the characteristic across the sequence.

A recurring theme in our research has been the paramount importance of the spatial context of an amino acid. We address this by incorporating 3D structural information into the visualization in two ways: (1) we use chords to connect the residues within a specified distance of a selected group of residues (thus identifying the "sphere of influence" of a region of the sequence); and (2) we include a 3D rendering of the functional protein.

## Related work

Sequence visualization tools [8] are most commonly employed to visualize genomes, rather than amino acid sequences. Some tools, like Artemis [9] visualize not only genomes, but also provide a higher-level view of a coding sequence and display amino acid properties, such as hydrophobicity. Common genome visualization tools like the *UCSC genome browser *[10] or *IGV *[11] use a track based approach, where multiple data sources are represented as one track each. In theory, such multiple tracks could be used to represent multiple amino acid indices, for all residues in a sequence concurrently. However, we chose to avoid a track based approach, since we intended to produce a more concise representation, and since we argue that such a representation does not adequately show situations where smaller effects in multiple tracks accumulate to a large overall effect.

The second class of visualization techniques related to Mu-8 are multiple sequence alignment visualization tools [12]. While Mu-8 does intentionally not show multiple sequence alignment, tools like VISSA [13] or PFATT [14] show not only the multiple aligned protein sequences but also provide some additional data, such as the predicted secondary structure, for the sequences. Both tools combine protein sequences with a 3D structure viewer.

Visualization of amino acid indices and protein sequences are, with the limited exceptions noted above, surprisingly rare. There are some visualizations, such as the one introduced by Bulka et al. [15] that show the properties of amino acids and their effects on substitution matrices in general. However, to our knowledge there is currently no approach that visualizes amino acid index data in general on a sequence, and no tool that visualizes the differences between protein sequences with respect to amino acid indices. Mu-8 was developed to address this shortcoming of current tools.

## Data and preprocessing

To use Mu-8, analysts have to provide two datasets: the sequence data of the reference protein and the protein family, and a file describing the 3D structure of a functional reference protein. In this paper we demonstrate Mu-8 using the defective *triose-phosphate isomerase (TIM) *sequence published as part of the BioVis Contest. TIM enzymes are utilized in glycolysis, an important metabolic process, and are essential for energy production. The enzyme is found in all living organisms and, in the case of humans, mutations can cause a severe metabolic disease called *triosephosphate isomerase deficiency*. The dataset contains a functioning TIM isolated from *Saccharomyces cerevisiae (scTIM) *[3], a family of functional TIMs, and a defective TIM (dTIM) created from mutating scTIM [2].

In addition to the data provided by the user, Mu-8 uses a set of amino acid indices from the *GenomeNet AAindex database *[16,17]. In this section, we elaborate on the pre-processing stage of the analysis.

### Sequence data

The amino acid sequence data for the proteins must be provided in an aligned format. The contest dataset includes dTIM (non-functional), scTIM (functional parent of dTIM), and a set of 5,508 other TIMs which we call the *family*. The length of both dTIM and scTIM is 248 residues, while other TIMs vary between 23 and 1053 with an average of 228 residues. To incorporate TIMs of different lengths, we conducted a multiple sequence alignment using the *Clustal *software [18]. Amino acids outside of the aligned residue window of the dysfunctional protein must be cropped off.

### 3D structure and proximity data

The 3D structure must be provided in the Protein Data Bank (pdb) file format. We demonstrate Mu-8 using the three-dimensional PDB model of scTIM [3]. Based on the supplied files, we compute pairwise distances between the a-carbons of each amino acid to determine whether two amino acids are within each other's sphere of influence.

### Index data and characteristic scores

Amino acid indices are quantitative measures of molecular characteristics. Mu-8 includes data on indices pertaining to six characteristics, for a total of more than 500 indices, originally analyzed by Tomii and Kanehisa [17]. These include:

• **alpha and turn propensity**, which quantifies the likelihood of forming an a-helix,

• **beta propensity**, which quantifies the likelihood of forming a b-sheet,

• **hydrophobicity**, which quantifies how water-repellent an amino acid is,

• **composition**, which quantifies the types of atoms that comprise each amino acid,

• **physicochemical properties**, which quantifies physical and chemical characteristics such as bulkiness, and

• **other properties**, which describes indices that do not fit within the other 5 categories, such as the likelihood that an amino acid will be located on the surface of the proteins.

An example index from the alpha and turn propensity group, developed by Prabhakaran [19], provides a score for the relative frequency of a residue in an alpha-helix structure, and is defined as the ratio of the observed to expected frequency of the residue in the alpha helix structure. Residues with greater than expected frequency have an index greater than one.

The large number of indices available can make the selection process difficult. We provide an alternative for analysts who either do not know which index to use or would like a single representative score for each of the six characteristics. To this end, we reduce the dimensionality of the indices using the method of principal components, for each of the six characteristics. For our sample data, we found that the first principal component accounts for a significant proportion of variability (between 50% and 75% for the 6 characteristics for the TIM data) which makes them reasonable representatives when faced with hundreds of indices from which to choose.

Based on either the first principal component of the indices, or the actual index values, we calculate a score, the c-score $cs_{ref}^{p,r}$, that quantifies the difference of the reference amino acid to the family, while also accounting for conservation. This score is calculated using the formula

(1) $$cs_{ref}^{p,r}=\frac{is_{ref}^{p,r}-is_{fam}^{\bar{p,r}}}{{\sigma_{is}}_{fam}^{p,r}},$$

where $is_{ref}^{p,r}$ denotes the index value or principal component of *p *for residue *r *of the reference sequence, $is_{fam}^{\bar{p,r}}$ denotes the average of *p *for residue *r *across the family, and ${\sigma_{is}}_{fam}^{p,r}$ is the standard deviation of the family's respective values.

The impetus for this metric is to identify locations of the sequence in which the amino acid index (or the principal component if that is being used) is significantly different from the family mean in positions that are highly conserved. Significantly high or low scores highlight residues of the reference protein that warrant further investigation.

## The Mu-8 Interface

In this section we discuss the design rationale for the visual encodings of the sequence, the c-scores, our measure of conservation, the 3D structure and the proximity data. In concert these provide the analyst with the desired holistic view.

### Score histograms

**Figure 2:**
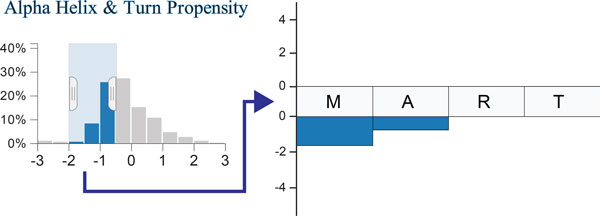
**Filtering of c-scores between -0.5 and -2 for the alpha helix & turn propensity characteristic**. An example of how such a score is mapped to the sequence is shown on the right.

The six histograms at the top of the visualization (see Figure 1) show the distributions of the c-scores, conveying the protein's difference to its family across the entire sequence. The tails of these distributions encode for residues that have either a significantly greater or smaller c-score than the family, i.e., the amino acids at the tails behave significantly different than the family. The histograms use a uniform y-axis and are capped at ±3 standard deviations to counter-balance the visual effects of outliers. The histograms can be used to filter scores in a selected range. Figure 2, for example, shows a filter excluding all scores outside the -2 to -0.5 interval. This is especially useful to select the tails of the distribution to highlight, for example, all amino acids that have a strongly increased hydrophobicity compared to the family consensus. Each histogram is given a unique color to identify the characteristics, which corresponds to the color of the bars in the sequence views. Regions of the histogram that are filtered-out are shown in gray.

The score histogram is also used to choose from the principal components of the six major characteristics, or from the full list of amino acid indices. By clicking the label above the histogram, a selection menu, containing a list of available characteristics and amino acids indices is revealed. Selecting an entry from the list replaces the data previously associated with the histogram with the selected entry's data.

### Sequence views

At the center of Mu-8 are two sequence views which are used to encode the c-scores and the degree of conservation of the residues. The *context sequence view *shows the whole sequence of amino acids from left to right. A labeled axis below the sequence facilitates orientation and enables analysts to easily reference regions.

Above and below the sequence we show stacked bars encoding the c-scores for each characteristic, thus highlighting the cumulative deviation from the family. Characteristics with a positive c-score are stacked on top of the sequence, while those with a negative score are stacked below the sequence. Figure 2 shows an example for the relationship of the histograms to the amino acid sequence. For the part of the sequence shown, two amino acids have scores matching the filter specified in the histogram, thus the corresponding bars are rendered.

While the context sequence view provides a convenient overview of the whole sequence, details such as the specific amino acid or the exact scores remain obscured. We therefore supplement the context sequence view with a *focus sequence view *also shown in Figure 1, which provides a larger version of a selected region of interest. The selected region is specified using a window on the context region, the size of which can be dynamically adjusted, but has an upper limit of 15 residues to ensure readability of the focus sequence.

**Figure 3:**
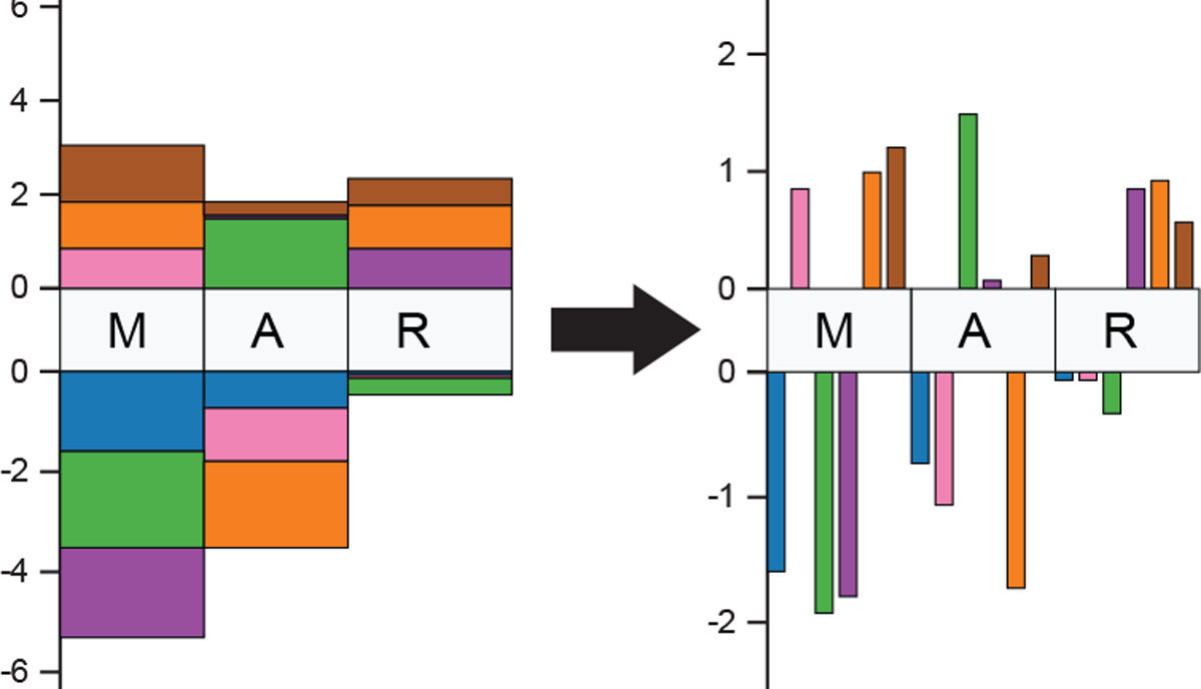
**Stacked bars compared to aligned bars for several residues**.

The stacked bars used in the context sequence allow an analyst to easily judge the overall deviation from the family. Judging the magnitude of the individual scores, however, is difficult using the stacked bars, as relative lengths of not-aligned elements are perceptually more difficult to distinguish compared to judging relative lengths of aligned elements, as shown in Figure 3. In the focus view, we provide the option to switch c-scores from a stacked to an aligned bar chart--which facilitates detailed comparisons within and amongst residues.

### Conservation heat map

Below the context sequence view is the *conservation heat map*, also shown in Figure 1. For each characteristic, this heat map encodes the variation of c-scores in the family. Conserved regions are known to be more relevant for function, since evolutionary pressure selects for functional proteins, while variable regions often are less relevant for function. As previously mentioned, conservation is also considered when calculating the c-scores, which results in higher scores for deviations in highly conserved regions. The additional heat map enables the analyst to judge conservation independently from effect size and judge the relevance of outliers. In the heat map dark cells encode a high variability, while bright cells encode for a conserved residue. Each row of the heat map corresponds to the variation of a characteristic's c-score. We encode the association of the rows to the c-scores using matching hues between the histograms and bars on the sequence and a consistent order: left-to-right in the histograms corresponds to top-to-bottom in the heat map. We use an HSL color scale to match the perceived brightness of the gray-scale and the colored areas.

### Visualizing proximity

Changes in the biochemical properties of the sequence influence the folding and thus the function of a protein. A linear representation of the amino acid sequence, however, cannot adequately account for the biochemical spheres of influence of the residues. Therefore we supplement the sequence view with proximity chords and provide a 3D structure view.

The *proximity chords *connect the focus region of the sequence with other residues that are within a user-specified distance from the focus region, as shown on top of the context sequence view in Figure 1. The sphere of influence that is of interest depends on the type of analysis. To account for this we provide the analyst with the means to specify the proximity using the *sequence proximity histogram*, shown at the lower right of Figure 1. This histogram shows the distribution of the distances of all residues relative to the residues in the focus region. By brushing the histogram, the analyst can specify the relevant proximity, which in turn filters the chords above the sequence. The chords are rendered at varying brightnesses, with darker chords encoding closer residues and brighter chords encoding more distant residues, as encoded in the legend above the histogram.

**Figure 4:**
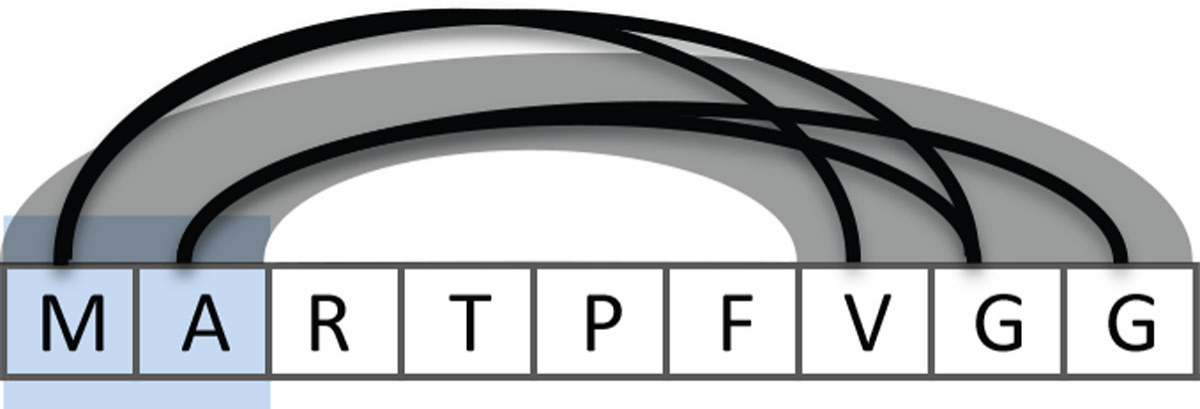
**The residues in the focus region are within the specified distance of three adjacent residues further down the sequence, as illustrated by the black arcs**. To reduce visual clutter, we replace the arcs connecting individual residues with chords (shown in gray) that connect proximate regions.

It is natural that the immediate neighborhoods of a residue are at similar distances to other neighborhoods in the sequence. We use this observation to reduce the visual clutter of the chords by bundling regions with similar proximity, as illustrated in Figure 4. In this example, the two residues in the focus region (M and A) are all connected to three residues adjacent to each other (V, G, and G). Instead of rendering a chord for every residue, as shown in black, we bundle them to a wider arch, shown in gray.

### Visualizing 3D structure

As the 3D structure is driving the function of the protein, it is a critical piece of information when analyzing a dysfunctional protein. As structural information for the whole family of proteins and the reference protein is typically not available, we limit our visualization to one, typically functional protein of the family. By linking the aligned sequence of the reference protein to the 3D structure, an analyst can identify which regions in the sequence coincide with the critical areas in the folded protein.

**Figure 5:**
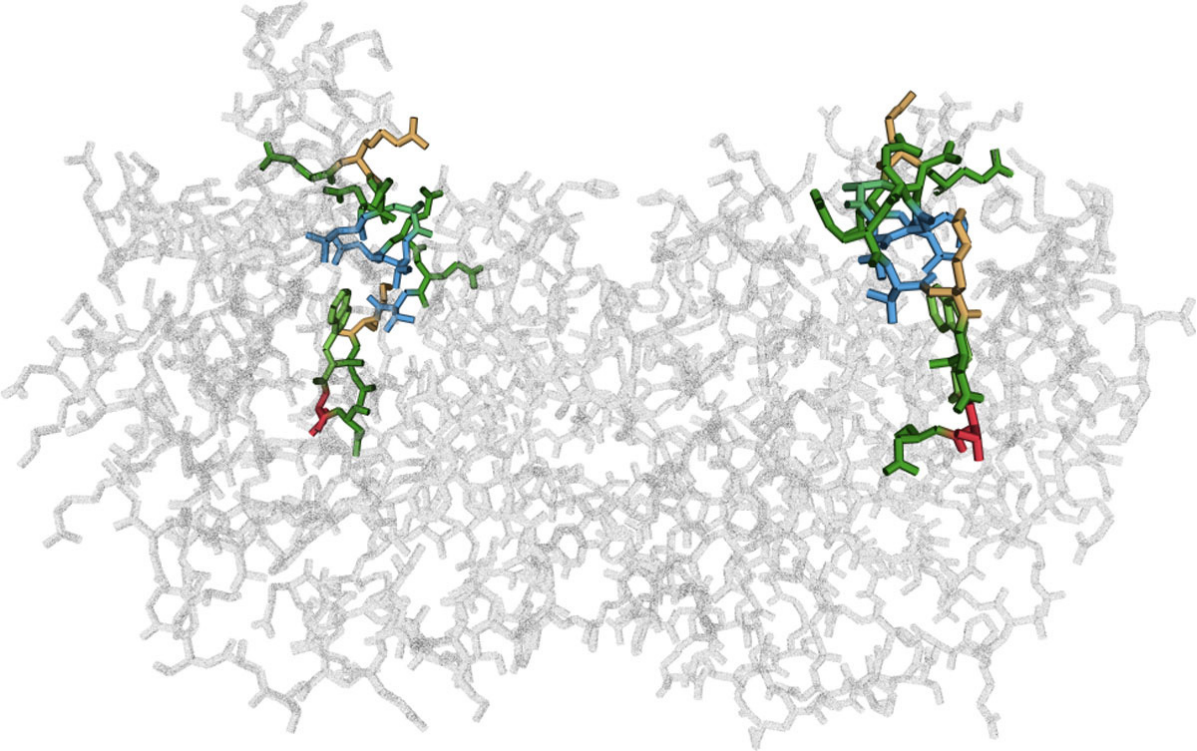
**3D all atom visualization of the folded protein**. The structure is linked to the sequence by color-coding the residues that are currently in the focus region of the sequence view.

We show the three-dimensional structure in an all-atom visualization (omitting hydrogen atoms), which we chose over a visualization of the secondary structure or the protein surface due to the residue centric paradigm of Mu-8. The structure view is shown at the bottom left of Figure 1 and in detail in Figure 5.

The view can be rotated, zoomed, and panned to inspect neighborhoods more closely. It is also linked to the sequence views such that the residues in the focus region are highlighted using an established color scheme for amino acids [20], and using stochastic order-independent transparency [21] for residues outside of the focus region.

## Implementation and scalability

We pre-processed the amino acid index data using R and C code. The visualization uses the D3 JavaScript library [22], with the exception of the 3D view, which employs WebGL. Mu-8 is open source, the code and data are accessible through the project website http://www.mu-8.com. We tested our implementation on recent versions of Google Chrome and Mozilla Firefox. Microsoft Internet Explorer currently does not support WebGL and thus cannot be used to run Mu-8.

The Mu-8 website enables biologists to provide their own MSA and PDB data for analysis. These datasets must be in a specified format and structural requirements are listed on the website. Registration and login are required for uploading datasets and enable persistence of data and future collaboration of analyses.

Mu-8 scales well to the requirements of most protein families. For humans, the median protein length is estimated to be in the 400-500 amino acid region [23]. Mu-8 handles proteins up to a length of approximately 1000 amino acids well. Beyond that an amino acid is represented by less than two pixels on a full-HD screen, limiting the usefulness of the approach. While this makes Mu-8 applicable to the majority of proteins, there are some that exceed this size considerably, which would require a modified approach.

## Conclusion and future work

We contend that Mu-8 is a comprehensive visual analysis solution to compare differences between a protein and its family. Our approach elucidates the significant biochemical differences while accounting for conservation, proximity amongst residues, and overall 3D structure. Mu-8 enables analysts to provide their own datasets and enables them to easily share visualizations with collaborators.

An interesting direction for future investigation is to integrate alignment data into Mu-8. Currently, Mu-8 does not consider sequence segments outside of the reference protein and also does not visualize gaps in the family that do not occur in the reference. Another area warranting research is to improve Mu-8's scalability, to also address the rare very large proteins. Here, approaches similar to genome browsers, with multiple levels of details, promise a solution.

As previously mentioned, Mu-8 reveals several candidate regions that may cause function to break down in the dTIM protein under consideration in the BioVis contest. The most notable mutated region is "LAGLGDE" located at positions 150-156. The evidence suggests that this region is: (1) significantly different across several characteristics, (2) relatively conserved, (3) close to other regions that exhibit suspect behavior in the folded protein, and (4) close to the proteins active site.

## Competing interests

The authors declare that they have no competing interests.

## Authors' contributions

JM, BP, AL, NB and HP jointly developed the concept and wrote the paper. JM, BP and NB developed the software, JM and AL elicited requirements from the domain experts.

## Declarations

Publication cost was supported in part by the Austrian Science Fund (J 3437-N15), the National Science Foundation (CGV-1111415) and the Air Force Research Laboratory and DARPA grant FA8750-12-C-0300.

This article has been published as part of *BMC Proceedings *Volume 8 Supplement 2, 2014: Proceedings of the 3rd Annual Symposium on Biological Data Visualization: Data Analysis and Redesign Contests. The full contents of the supplement are available online at http://www.biomedcentral.com/bmcproc/supplements/8/S2
